# Size-dependent Nanoparticle Accumulation In Venous Malformations

**DOI:** 10.1097/JOVA.0000000000000103

**Published:** 2024-12-06

**Authors:** Kathleen Cullion, Claire A. Ostertag-Hill, Weimin Tang, Michelle Pan, Daniel S. Kohane

**Affiliations:** a Laboratory for Biomaterials and Drug Delivery, Boston Children’s Hospital, Harvard Medical School, Boston, Massachusetts; b Department of Medical Critical Care, Boston Children’s Hospital, Harvard Medical School, Boston, Massachusetts; c Department of Surgery, Boston Children’s Hospital, Harvard Medical School, Boston, Massachusetts; d Department of Anesthesiology, Critical Care, and Pain Management, Boston Children’s Hospital, Harvard Medical School, Boston, Massachusetts

**Keywords:** drug delivery, enhanced permeation and retention, nanoparticles, venous malformations

## Abstract

**Objective:**

The current treatment of venous malformations (VMs) consists of medications with systemic toxicity and procedural interventions with high technical difficulty and risk of hemorrhage. Using nanoparticles (NPs) to enhance drug delivery to VMs could enhance efficacy and decrease systemic toxicity. NPs can accumulate in tissues with abnormal vasculature, a concept known as the enhanced permeation and retention (EPR) effect. EPR has been documented in tumors, bioengineered vessels, and VMs. However, in VMs, it is unknown if NP size affects EPR and if so, which particle size improves NP accumulation.

**Methods:**

In this study, we used a murine model of subcutaneous VMs using human umbilical vein endothelial cells that express the most frequent VM-causing tyrosine kinase with immunoglobulin and EGF homology domains mutation, tyrosine kinase with immunoglobulin and EGF homology domains-L914F. Hollow silica NPs coated in polyethylene glycol (PEG) and conjugated to a fluorophore were administered systemically via tail vein injection. We studied the accumulation of a range of NP sizes within the VM and organs using confocal microscopy and an in vivo imaging system.

**Results:**

The 20, 50, 80, and 180 nm PEGylated, fluorophore-tagged hollow silica NPs were spherical and had hydrodynamic diameters of 31.6 ± 0.9, 58.5 ± 0.1, 87.1 ± 2.4, and 232 ± 1.26 nm, respectively. Following systemic NP administration, 20 nm NPs had 2 times more fluorescence accumulation within VMs compared with 50 nm, and 6 times more fluorescence accumulation compared with larger (greater than 80 nm) NPs (*P* < .01).

**Conclusion:**

This study helps to determine the optimal NP size for passive accumulation within VMs and lays the foundation for engineering NPs for the treatment of VMs.

## Introduction

Venous malformations (VMs) are slow-flow vascular lesions comprised of ectatic endothelial cell-lined channels covered by irregularly distributed smooth muscle cells. Depending upon the location, VMs can be associated with disfigurement and functional impairment, whereas localized coagulation within the malformation can cause pain and inflammation.^1^ Current treatment of VMs depends on the anatomical location and often includes repeated invasive procedures (including sclerotherapy and if technically possible, surgery)^2,3^ and life-long pharmacotherapy that can be associated with side effects including immunosuppression (with increased risk of infection) and common toxicities such as mouth sores, anemia, and gastrointestinal upset.^4^

When free drugs are delivered intravenously or orally, only a small fraction reaches the intended target. Most of the drug accumulates in healthy tissue, causing side effects, as seen in children receiving life-long systemic sirolimus therapy.^4^ Encapsulating drugs within nanoparticles (NPs) can improve drug delivery to specific target tissues and may be associated with decreased side effects. (The majority of NPs still accumulate off-target but the proportion on-target increases, improving the therapeutic index.)^5^ When administered systemically, nanosized agents may preferentially accumulate in organs with abnormal (“leaky”) vasculature, a concept known as the enhanced permeation and retention (EPR) effect.^6^ EPR or EPR-like effects have been documented in tumors,^7^ choroidal neovascularization,^8^ myocardial ischemia,^9^ and bioengineered vessels.^10^ We have previously demonstrated the accumulation of systemically administered gold NPs within VMs via EPR-like effects.^11^

Many factors affect NP accumulation within target tissues including physicochemical properties of the NP (size, shape, flexibility, surface charge, and composition), the tissue (leakiness of the vasculature and rate of blood flow), and the distribution, systemic circulation, and elimination method of the NP in the biological system.^12^ In solid tumor models, NP sizes between 10 and 250 nm are optimal for EPR.^6,13–16^ However, the exact size of NPs that will promote optimal NP accumulation within VMs is unknown.

Here, we report the size-dependent EPR effect of systemically administered PEGylated silica NP standards on passive accumulation within VMs. Importantly, we use a VM model derived from human umbilical vein endothelial cells (HUVECs) induced with a tyrosine kinase with immunoglobulin and EGF homology domains (TIE2)-activation mutation (L914F). These cells (HUVEC-TIE2-L914F) form VM lesions in immunodeficient mice and the model accurately reflects the biology of VMs in humans.^17^

## Methods

### Reagents

Methoxypolyethylene glycol succinimidyl ester (mPEG-NHS) with a molecular weight of 2000 was purchased (Nanocs, New York, New York). Aminated silica nanospheres (20, 50, 80, and 180 nm) were purchased (nanoComposix, San Diego, California). Dimethyl sulfoxide (DMSO) and fetal bovine serum were purchased (Sigma-Aldrich, St. Louis, Missouri). Geltrex LDEV-Free Reduced Growth Factor Basement Membrane Matrix and AlexaFluor-647 NHS succinimidyl ester were purchased (Thermo Fisher Scientific, Waltham, Massachusetts). Endothelial basal medium and endothelial cell growth medium singlequots supplements were purchased (Lonza, Basel, Switzerland). Fluorescein labeled 4′,6-diamidino-2-phenylindole (DAPI), and Ulex Europaeus Agglutinin I (UEA I) were purchased (Vector Laboratories, Newark, California).

### Preparation of Pegylated, Fluorophore-Conjugated Hollow Silica Nanoparticles (ie, HSNP-647)

Silica nanospheres were purchased with an aminated surface and conjugated to mPEG-NHS-2k and AlexaFluor-647 NHS Ester as follows. The desired amount of HSN (suspended in ethanol) is sonicated for 5 minutes prior to use. mPEG-NHS-2k is dissolved in DMSO (0.15 mg/μL) and added to HSN for a final concentration of 30 mg mPEG/mL HSNP. AlexaFluor-647, dissolved in DMSO, was added for a final concentration of 4 w/w%. These materials were incubated overnight at 4°C while stirring. The formulations were then dialyzed in deionized water for 72 h, followed by dialysis in 1X PBS for 48 h at 4°C to remove ethanol and any unreacted reagents. Formulations were concentrated to 5 mg/mL by gentle ultracentrifugation and stored at 4°C until use.

### ^1^H NMR Spectroscopy

A total of 5 mg of each HSN and HSNP formulation (20, 50, 80, and 180 nm) were suspended in 600 μL of DMSO-D6 and assessed on a Varian 400 M nuclear magnetic resonance (NMR) spectroscope. The spectra were referenced to the residual solvent peak in DMSO at 2.48 and 3.29 ppm for ^1^H proton.

### Ultraviolet-Visible Spectroscopy

To assess the conjugation of Alexa-647 to HSNs, the ultraviolet-visible absorption spectra (200–800 nm) 5 mg/mL of unconjugated HSNs and conjugated HSNP-647 were measured by spectrophotometry (Agilent Cary UV-Vis-NIR spectrophotometer, Santa Clara, California). Samples were diluted in DMSO.

### Transmission Electron Microscopy

Each formulation of HSNP-647 (5 μL) was deposited on a copper grid coated by a carbon film, washed, then stained with uranyl formate (5 μL). The sample was dried and imaged using a Tecnai G^2^ Spirit BioTWIN transmission electron microscope (FEI Company, Hillsboro, Oregon) operating at 80 kV.

### DLS and Zeta Potential

Particle size and zeta potential were measured by a Malvern Nano ZetaSizer (Malvern Panalytical, Westborough, Massachusetts).

### Biodistribution of HSNP-647 Following Systemic Injection

Male 6–8-week-old nude athymic (nu/nu) mice were purchased (Massachusetts General Hospital, Boston, Massachusetts). All experimentation was conducted in accordance with protocols approved by Boston Children’s Hospital Institutional Animal Care and Use Committee. Human umbilical vein endothelial cells (HUVECs) with a mutation in the angiopoietin-1 receptor (TIE2, TIE2-L914F) (abbreviated HUVEC-TIE2-L914F) were cultured on 0.1% gelatin-coated plates with endothelial basal medium supplemented by endothelial cell growth medium singlequots supplements and 10% fetal bovine serum. HUVEC-TIE2-L914F cells were obtained directly from Dr. Elisa Boscolo laboratory.

Murine VM model was generated as described.^17^ Briefly, cells (2.5 × 10^6^ HUVEC-TIE2-L914F) were isolated and suspended in Geltrex LDEV-Free Reduced Growth Factor Basement Membrane Matrix. For each VM, 200 mL of the cell suspension was injected subcutaneously with a 26G needle into the dorsal flank of the animal. Mice were monitored for the days following the injections and the size (length and width) of the VMs were measured each day with a digital caliper. VM volume was calculated 1/2 l × w2.

To determine the biodistribution of NPs, mice (n = 4) were injected (intravenously) with each formulation of HSNP-647 (5 mg/mL) or saline once VM volumes reached 300 mm^3^ ± 100 mm^3^. At 24 hours postinjection, animals were sacrificed, and VMs and organs were harvested and imaged ex vivo with an in vivo imaging system (IVIS, Lumina Series III, Perkin Elmer, Massachusetts) using the following settings; excitation: 580–660 nm, emission: 660–710 nm. Fluorescence intensity was quantitated as total radiant efficiency (TRE, the emission radiance divided by excitation light power, [photons/s/cm^2^/sr]/µW/cm^2^), using LivingImage (Perkin Elmer). TRE was normalized by dividing it by the peak absorption at 662 nm for a given particle size, and then by the fluorescent signal following saline injection. The relative masses of particles that accumulated in tissues per gram of tissue were compared by dividing the normalized TRE by tissue mass. Animals were fed a Verified 75 IF Irradiated diet (ScottPharma, Marlborough, Massachusetts) before experimentation. Following IVIS imaging, the VMs were prepared for histology.

### Histology

VMs were snap frozen in optimal cutting temperature compound. Samples were sent to iHisto (Salem, Massachusetts) and were stained with hematoxylin and eosin, 4′,6-diamidino-2-phenylindole, or with UEA I using standard staining procedures. Bright-field and fluorescent images were obtained using the Zeiss Axio Imager. Tiling and stitching were performed using Zeiss ZEN software.

### Statistical Analyses

All data shown are expressed as the mean ± standard deviation. All *P*-values were calculated by the one-way analysis of variance with post hoc Tukey honestly significant difference test. *P* <.05 was considered statistically significant. Statistical analysis was conducted using GraphPad Prism (version 9.5.1) (Boston, Massachusetts).

## Results

### Preparation and characterization of PEGylated, fluorophore-conjugated hollow silica nanoparticles

**Figure 1.**
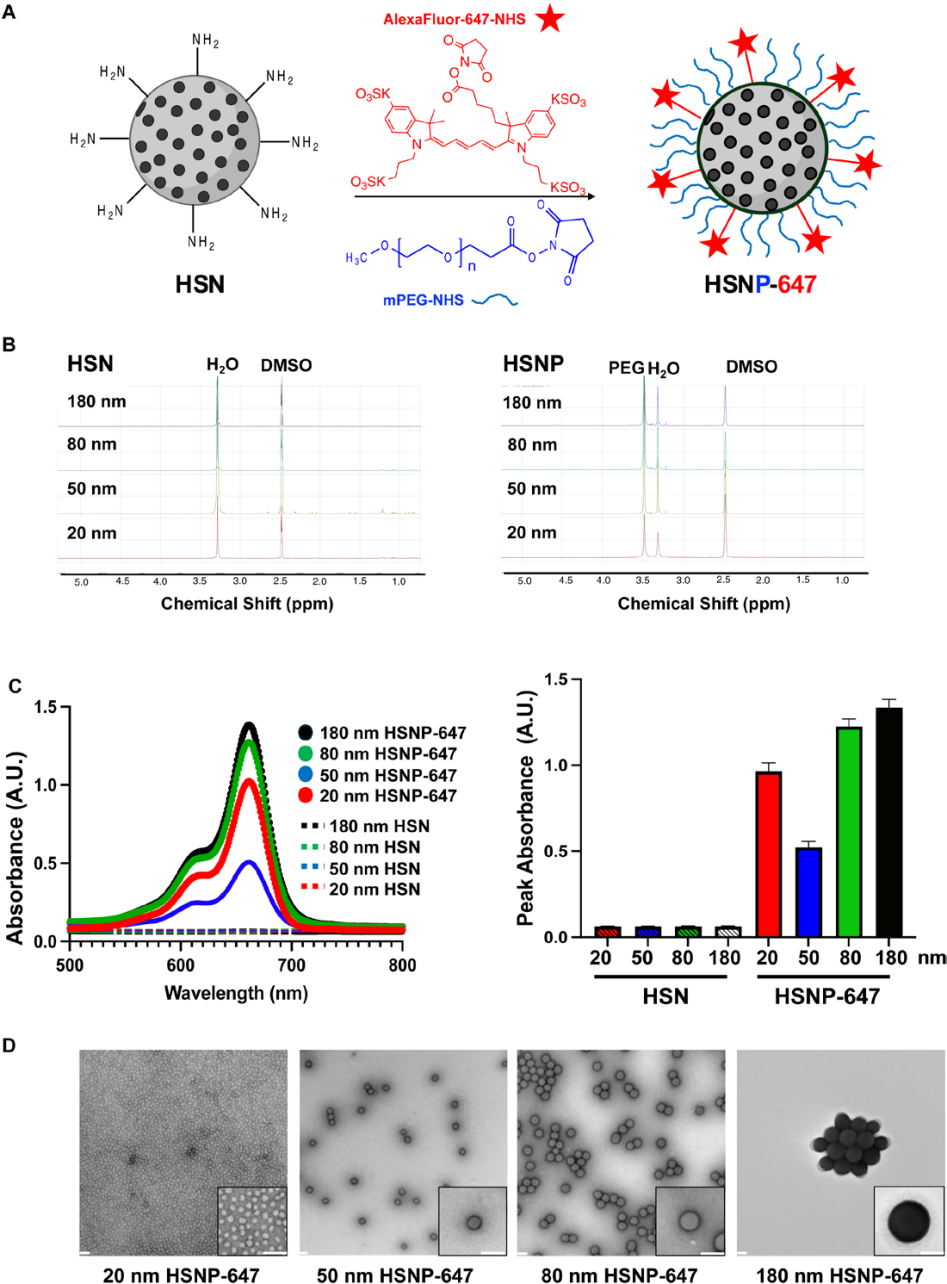
Characterization of NPs. (A) Scheme of aminated hollow silica NP (HSN) conjugated to polyethylene glycol (PEG) and fluorophore (Alexa-647 NHS) to make HSNP-647. (B) ^1^H-NMR spectroscopy prior to (HSN) and after (HSNP) 2000 Da poly(ethylene glycol) (PEG) conjugation. (C) Absorbance spectra (from 500 to 800 nm) and peak absorbance at 662 nm on ultraviolet-visible spectroscopy, prior to (HSN) and after (HSNP-647) Alexa-647 conjugation. (D) Characterization of HSNP-647 by transmission electron microscopy (TEM) (scale bar 100 nm). HSN indicates hollow silica nanoparticle; HSNP, PEGylated hollow silica nanoparticle; HSNP-647, Alexa-647-labeled HSNP; NMR, nuclear magnetic resonance.

To determine which NP size will have the greatest accumulation within VMs, we studied 4 particle sizes in the range of 20–250 nm, a range of sizes that has been shown to promote EPR in models of cancer, bioengineered tissues, myocardial infarction, tuberculosis, and choroidal neovascularization.^7–10,12^ We studied hollow silica NP standards (HSNs) because of their inert, nontoxic nature, narrow size distribution, and surface chemistry allowing surface modification.^18^ The aminated HSN surfaces were modified with 2000 Da poly(ethylene glycol) (PEG) (PEGylated HSN are abbreviated HSNP) to extend their circulation time^19^ and then labeled with a fluorophore (Alexa-647) for in vivo tracking^18^ (PEG-modified and Alexa-647-labeled HSNPs are abbreviated HSNP-647) (Figure 1A). Alexa-647 was chosen because its excitation/emission spectra (excitation: 580–660 nm, emission: 660–710 nm) are distinct to those of red blood cell autofluorescence, and will allow tracking of NPs in vivo with an in vivo imaging system (IVIS), and study of the distribution of NPs within VMs using confocal microscopy.

^1^H-nuclear magnetic resonance spectroscopy confirmed the conjugation of PEG to the surface of HSNs, creating HSNPs (Figure 1B). Ultraviolet-visible spectroscopy confirmed the conjugation of Alexa-647 to the surface of HSNP-647s by the presence of a peak at 662 nm. Since the same mass (5 mg) of each particle size was used, the differences in the height of that peak (Figure 1C) were used to normalize downstream data to determine the relative masses of particles that accumulated in tissues.

Transmission electron microscopy after modification confirmed that all HSNP-647s were spherical (Figure 1D) and determined particle diameters to be 20.2, 50.2, 80.4, and 180 nm, respectively (Table 1) (for simplicity, particles will be referred to as 20, 50, 80, and 180 nm, the sized provided by the manufacturer). The hydrodynamic diameter, polydispersity index, and zeta potential of particles pre (HSN) and post (HSNP-647) PEG/Alexa-647 conjugation were determined by dynamic light scattering (Table 1). In all cases, the diameter by dynamic light scattering was larger than that measured by transmission electron microscopy, because the former measures hydrodynamic diameter which takes into account the water shell around the particle.^20^ For all formulations, the hydrodynamic diameters increased following the conjugation of PEG/Alexa-647. The polydispersity indices of the HSNPs were in the range of 0.05–0.23, reflecting a narrow size distribution. (Polydispersity is a measure of size heterogeneity, ranging from 0.0 [perfectly uniform] to 1.0 [highly polydisperse].) Zeta potentials of the HSNP-647s were in the range of −1.5 to −5 mV, reflecting a relatively neutral charge. (Zeta potential is a measure of the net particle surface charge. Zeta potentials less than −30 mV and greater than +30 mV are considered strongly anionic or cationic, respectively.) The change from the more positive charge of the HSNs reflects the change from an aminated surface to a surface decorated with PEG/Alexa-Fluor (Table 1).

**Table 1.** NP Characterization

HSN	20 nm	50 nm	80 nm	180 nm
Diameter from manufacturer^a^ (nm)	20	50	80	180
Hydrodynamic diameter (nm)	31.2 ± 4.55	54.8 ± 1.16	82.4 ± 1.76	208 ± 4.59
Polydispersity index	0.213 ± 0.01	0.029 ± 0.01	0.018 ± 0.01	0.08 ± 0.05
Zeta potential (mV)	17.2 ± 0.23	7.9 ± 1.36	25.1 ± 0.28	23.5 ± 1.86
**HSNP-647**	**20 nm**	**50 nm**	**80 nm**	**180 nm**
Measured diameter TEM (nm)	20.2	50.2	80.4	180.0
Hydrodynamic diameter (nm)	37.7 ± 0.9	58.5 ± 0.1	87.1 ± 2.4	232 ± 1.26
Polydispersity index	0.232 ± 0.01	0.16 ± 0.02	0.08 ± 0.01	0.05 ± 0.02
Zeta potential (mV)	−4.35 ± 1.5	−1.5 ± 0.6	−4.99 ± 1.4	−4.1 ± 0.8

Abbreviation: TEM, transmission electron microscopy.

a Determined by TEM by the manufacturer.

### Generation of venous malformations in mice

Up to 60% of human VMs are associated with activated TIE2 mutations (the most common being p.L914F). This activating TIE2 mutation (p.L914F) is sufficient to induce HUVECs (HUVEC-TIE2-L914F) to form VM lesions in immunodeficient mice.^17^ In this model, TIE2-L914F-expressing HUVECs are suspended in Matrigel and injected into immunocompromised mice. Within 10 days, these mice develop vasculature that expands with similar characteristics to VMs described in patients.^17^ These new vessels are fully connected with the host vasculature, allowing for the study of systemically administered therapeutics.^11,17,21^

**Figure 2.**
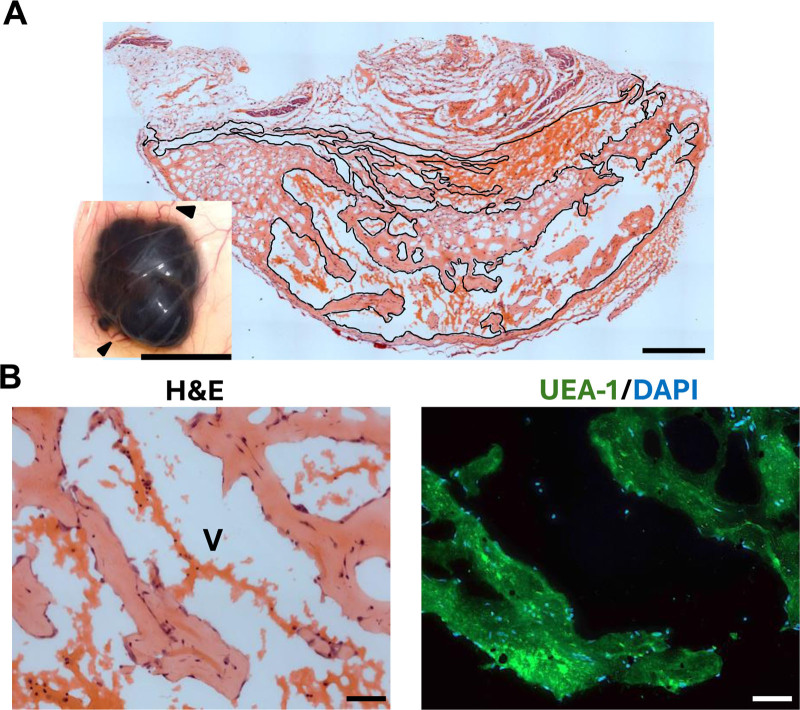
Murine model of venous malformations in mice. (A) Representative 150× image of hematoxylin and eosin (H&E) stained section of the VM. Perfused vessels are outlined in black. Scale bar: 1000 µm. Histological stitching performed with Zeiss ZEN software. The inset is a representative photo of a subcutaneous VM in situ with skin reflected. Arrows: host feeder vessels. Scale bar: 15 mm. (B) Detail of VM at high power magnification (600×). V: perfused vessel. H&E and immunohistochemical (nuclear stain: DAPI [blue], venous endothelium: UEA-1 [green]) staining of the vascular network. Scale bar: 50 µm. DAPI indicates 4′,6-diamidino-2-phenylindole; UEA-1, ulex europaeus agglutinin I; VM, venous malformation.

After culture in vitro, TIE2-L914F-expressing HUVECs were injected subcutaneously into mice as previously described,^17^ formed VMs. Once the VMs reached approximately 300 mm^3^, animals were euthanized and VMs were harvested. Upon dissection of the VMs and reflection of the skin, feeder vessels were identified (Figure 2A; inset, black arrows indicate feeder vessels) indicating anastomoses to the host vasculature. VMs had a median size of 283.8 mm^3^ ± 92.2 (n = 8). Immunohistochemical staining of the explants revealed a network of perfused blood vessels (Figure 2A), that is vessels containing red blood cells. The endothelia in the VMs stained positively with human ulex europaeus agglutinin I (UEA-1; green) (Figure 2B), indicating vessels comprising cells of human origin.^17^

### Accumulation and retention of HSNPs in VMs following systemic injection

**Figure 3.**
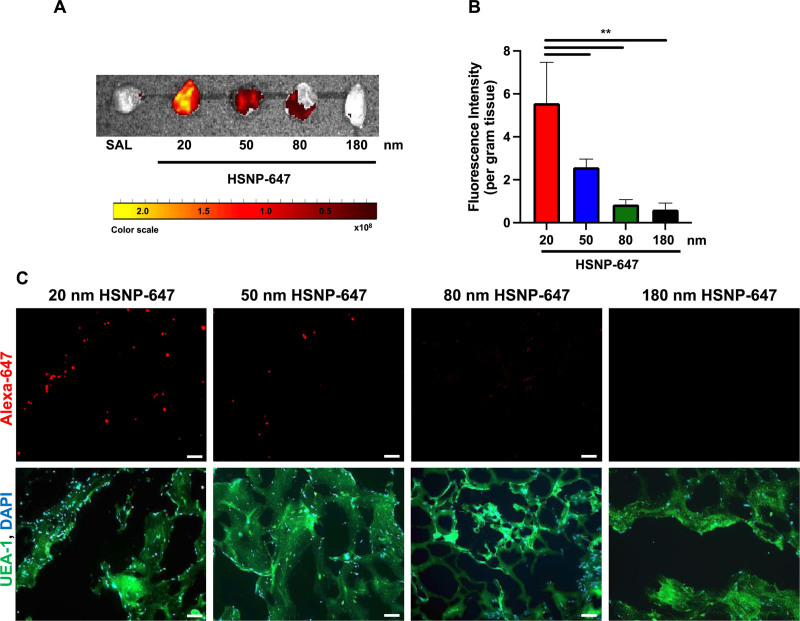
Fluorescence detected in VMs 24 hours following intravenous injection with saline (SAL) or 20, 50, 80, and 180 nm HSNP-647 (5 mg/ml). (A) Representative in vivo imaging systems (IVIS) image of VM ex vivo. (B) Fluorescence intensity per gram tissue in VMs normalized to saline control and to the measured peak absorbance of each particle. Data are means ± SD (n = 4) (*P* < .01). (C) Top panel is representative immunohistochemical (Alexa-647; red) staining of VMs, indicating HSNP-647 accumulation in VMs. Bottom panel is merged images of nuclear stain, DAPI (blue) and venous endothelium, human UEA-1 (green). DAPI indicates 4′,6-diamidino-2-phenylindole; HSNP, PEGylated hollow silica nanoparticle; HSNP-647, Alexa-647-labeled HSNP; UEA-1, ulex europaeus agglutinin I; VM, venous malformation.

Once the VMs reached approximately 300 mm^3^, 0.6 mg (in 200 µl) of 20, 50, 80, or 180 nm HSNP-647 or saline were injected via tail vein into animals with VMs. We have shown that after systemic injection of particles <50 nm, very little particle is detected in the blood by 24 hours.^10,11^ Therefore, we harvested VMs (and organs) 24 hours following injection with HSNP-647. An IVIS was used to measure the fluorescence of HSNP-647 in the harvested VMs, ex vivo (excitation: 580–660 nm, emission: 660–710 nm) (Figure 3A). To account for tissue autofluorescence, following injection of each HSNP-647 formulation, the fluorescence detected in VMs was normalized to fluorescence in saline-injected controls. To account for tissue mass, the fluorescence detected was normalized to VM weight and presented as fluorescence per gram of tissue. The fluorescence in VMs of animals injected with 20 nm HSNP-647 was 5-fold more than that in VMs of saline controls, 2-fold greater than in the VMs of animals injected with 50 nm HSNP-647s, and 6-fold greater than in the VMs of animals injected with larger (80 or 180 nm) HSNP-647 (*P* < .01) (Figure 3B). The fluorescence in VMs of animals injected with 50 nm HSNP-647 was greater than 2-fold more than that in VMs of animals injected with larger (80 or 180 nm) HSNP-647s (Figure 3B).

We confirmed these results by confocal microscopy, which showed the greatest fluorescence in VMs from animals that had been injected with 20 nm HSNP-647 compared with animals injected with larger (>50 nm) HSNP-647s (Figure 3C).

### Distribution of HSNP-647s following systemic injection

**Figure 4.**
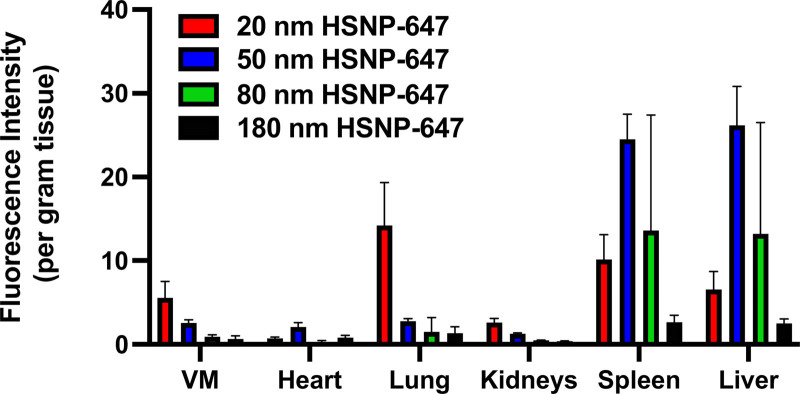
Size-dependent HSNP-647 distribution 24 hours following systemic injection of saline or fluorescently labeled HSNPs. Each color represents a different HSNP-647 size (red: 20 nm, blue: 50 nm, green: 80 nm, and yellow: 180 nm HSNP-647). Fluorescence intensity (per gram tissue) was measured for VMs and each organ and then normalized to saline control and to the measured peak absorbance of each particle. Data are means ± SD (n = 4). HSNPs indicates PEGylated hollow silica nanoparticles; HSNP-647, Alexa-647-labeled HSNP; VM, venous malformation.

To determine if NP size affects HSNP-647 biodistribution to various organs, whole organs were evaluated by IVIS, ex vivo, 24 hours following injection of 20, 50, 80, and 180 nm HSNP-647 or saline. Fluorescence per gram of tissue was highest in the spleen and the liver, regardless of HSNP size (Figure 4), as is the case with many other NP systems.^14,22^ Three-fold more fluorescence was detected in the lungs with 20 nm HSNP-647 than with larger (>50 nm) HSNP-647s (Figure 4). Minimal fluorescence was detected in the heart or kidney regardless of HSNP-647 size.

## Discussion

In this study, we have demonstrated the size-dependent accumulation of HSNP-647 within VMs through EPR-like effects. In this model of human VMs in mice, the greatest accumulation of HSNP-647s occurred with the smallest size NPs studied (20 nm and—to a lesser degree—50 nm).

All 4 formulations tested here were made of the same material, were coated with PEG, had a spherical shape, and a neutral surface change. Therefore, it is likely that the differences in particle accumulation between formulations were due to particle size rather than particle material, charge, or shape.

As in other systems,^14,22,23^ smaller particles had better accumulation in VMs. Not all particles can be made in the size range that we have found here to be best for accumulation in VMs. This might not be a problem for noble metal NPs such as gold nanoshells that might be used for thermal ablation of VMs,^11^ or micelles (NPs formed from self-assembly of amphipathic polymers and similar materials) to deliver drugs.^10^ However, liposomes (particles formed from lipid bilayers) or polymeric microspheres (ie, particles that are monolithically composed of a single polymer) are difficult to make <100 nm.^24^ Consequently, there may be a trade-off between the particle size that leads to the greatest accumulation of particles, and other parameters. Larger particles tend to have better drug loading and release drug more slowly, which may offset the fact that they accumulate to a lesser degree. It is also easier to encapsulate combinations of drugs with differing physicochemical properties in some of the larger particles.^25^ For example, liposomes have both hydrophobic domains (the lipid bilayers) and hydrophilic ones (the internal aqueous phase), and so can encapsulate both kinds of compounds.^26^ Smaller NPs also have the advantage that their accumulation can be enhanced to a greater degree by a variety of targeting strategies to further enhance accumulation in tissues.^8,10,27^

Drugs used to treat VMs have been nanoencapsulated.^28^ Different types of particles may be better for different types of drugs depending on the latter’s size and hydrophobicity. Here, the smallest NPs accumulated in VMs to the greatest degree. Particles of that size can deliver effective amounts of drug. For example, 15 nm micelles delivered the local anesthetic drug bupivacaine for intravenous regional local anesthesia,^29^ and 19 nm micelles delivered doxorubicin to treat choroidal neovascularization.^8^

Nanoencapsulation could increase the fraction of a given dose that accumulates in VMs. Whether that increase in accumulation would be clinically significant in terms of efficacy and toxicity (ie, therapeutic index) would depend on the degree to which the accumulation of any given NP was enhanced and the therapeutic index of the drug itself. Nanoencapsulation is well known to have these desirable effects in cancer,^6,30^ albeit with some controversy as to the extent to which the EPR effect occurs in humans.^31^

Novel treatments are needed not only for VMs but for many types of vascular anomalies. Utilizing nanoparticulate therapeutics and optimizing accumulation in VMs, as shown in this work, has the potential for broader application to other vascular anomalies. EPR occurs in tumors because of the structural and functional abnormalities of the composing blood vessels, such as deficient basement membranes, deficient smooth muscle cells, elevated inflammatory markers, and lack of efficient lymphatic drainage.^31^ On histopathologic analysis, VMs have ectatic, thin-walled venous channels lined by endothelia and surrounded by irregularly distributed smooth muscle cells.^32,33^ Several of these characteristics, in addition to the slow flow through VMs, appear to allow EPR to occur and thereby permit NP accumulation. Several other types of vascular anomalies share characteristics that might make them amenable to this approach. For example, capillary malformations, another type of slow-flow malformation, consists of thin-walled, ectatic, and dilated capillaries with flat endothelial cells and defective smooth muscle expression within pericytes,^34–36^ which could enable EPR. Combined vascular malformations, such as lymphatic-venous malformations or capillary-venous malformations, could also allow this approach. Further investigation is warranted to examine the broader applicability of the approach used in this work.

Here, silica NPs were selected for this proof-of-principle determination of the effect of particle size on accumulation in VMs. Given that material, charge, shape, and particle flexibility can affect distribution and accumulation, it is possible that the optimal size for HSN is not the optimal size for other types of particles. Characteristics of the target tissue (leakiness of the vasculature, rate of blood flow) could also affect the ideal particle size for NP accumulation (eg, in other types of vascular malformations). The type and size of nanoparticulate vehicle to be used will depend on the drug to be delivered and other factors. Each will have its own rate of degradation, and toxicity to organs, if any. Note that in general NP used in applications of this type have minimal or no toxicity. A separate consideration for the study is whether the accumulation of encapsulated drugs in organs will lead to toxicity.

This study determined the optimal NP size for passive accumulation of hollow silica NPs within VMs. This study lays the foundation for engineering NPs for the treatment of VMs, balancing therapeutic drug loading and passive selective accumulation based on the EPR effect.
